# Investigation on Neurobiological Mechanisms of Dreaming in the New Decade

**DOI:** 10.3390/brainsci11020220

**Published:** 2021-02-11

**Authors:** Serena Scarpelli, Valentina Alfonsi, Maurizio Gorgoni, Anna Maria Giannini, Luigi De Gennaro

**Affiliations:** 1 Body and Action Lab, IRCCS Fondazione Santa Lucia, 00179 Rome, Italy; valentina.alfonsi@uniroma1.it (V.A.); luigi.degennaro@uniroma1.it (L.D.G.); 2 Department of Psychology, Sapienza University of Rome, 00185 Rome, Italy; maurizio.gorgoni@uniroma1.it (M.G.); annamaria.giannini@uniroma1.it (A.M.G.)

**Keywords:** dream recall, sleep, EEG, neuroimaging, brain stimulation, activation, continuity

## Abstract

Dream research has advanced significantly over the last twenty years, thanks to the new applications of neuroimaging and electrophysiological techniques. Many findings pointed out that mental activity during sleep and wakefulness shared similar neural bases. On the other side, recent studies have highlighted that dream experience is promoted by significant brain activation, characterized by reduced low frequencies and increased rapid frequencies. Additionally, several studies confirmed that the posterior parietal area and prefrontal cortex are responsible for dream experience. Further, early results revealed that dreaming might be manipulated by sensory stimulations that would provoke the incorporation of specific cues into the dream scenario. Recently, transcranial stimulation techniques have been applied to modulate the level of consciousness during sleep, supporting previous findings and adding new information about neural correlates of dream recall. Overall, although multiple studies suggest that both the continuity and activation hypotheses provide a growing understanding of neural processes underlying dreaming, several issues are still unsolved. The impact of state-/trait-like variables, the influence of circadian and homeostatic factors, and the examination of parasomnia-like events to access dream contents are all opened issues deserving further deepening in future research.

## 1. Introduction

Dreaming, or sleep mentation, is an intriguing experience occurring during any human sleep stages [1]. It can happen many times per night and is characterized by different degrees of emotional intensity, bizarreness, visual vividness, and narrative complexity. Although dreaming is impossible to study directly, many researchers investigated its neural bases in healthy subjects [2,3,4,5,6,7,8,9,10,11,12,13,14] or clinical samples [15,16] in different age ranges [10,17,18]. Most investigations focused on dream recall (DR), namely the individuals’ content reported immediately after awakenings. However, several methods were applied to study dreaming, from naturalistic investigations using perspective diaries or retrospective questionnaires to psychophysiological and neuropsychological approaches.

The first studies on neural bases of dreaming were carried out on brain-lesioned patients to understand anatomical correlates of dream disappearance or excesses [19]. Overall, these early findings suggested that two brain networks are engaged in the oneiric processes: a posterior and an anterior system. Specifically, lesions concerning the right and unilateral posterior networks can affect some dream aspects, namely color or motor features [19]. Temporo-parietal junction (TPJ)-responsible for visual imagery during the waking state- is also involved in this system.

A lesion over the bilateral anterior system causes dream cessation, i.e., the so-called “anoneria” or Charcot–Wilbrand syndrome [19]. The ventromedial prefrontal cortex is included in this network and is involved in mental representation in wakefulness. In addition, many of the afferent and efferent fibers of these regions are linked to the limbic system, recognized as essential for dreaming [20]. Prefrontal leucotomy is also linked to dream loss, while lesions of the medial prefrontal cortex (mPFC) result in excessive dreaming, such as the so-called “anoneirognosis” [19].

These neuropsychological observations on brain lesioned patients enhanced our knowledge about the structures involved in dream experience. However, these studies are not free from flaws because (a) DR in patients could be affected by pharmacological treatments; (b) DR was retrospectively collected; (c) no information was available about DR during premorbid conditions [21].

Moreover, the research on the neural bases of sleep mentation is always interwoven with the issue concerning the functional role of dreaming. Oneiric experience has been considered a simulation of reality offering a coping strategy to face daily adverse events [22]. More in general, it could represent an emotional regulator and some neurobiological correlates provide support to this view [23]. In this vein, some authors highlighted that daily experience could be incorporated and re-organized during dreaming. For instance, pre-sleep rehearsal of actual concerns and problems before sleeping can appear in DR [24,25].

New techniques have been introduced to obtain a deeper understanding of the neurobiological processes underlying dreaming starting from these early studies. Furthermore, in a pioneering way, various studies were aimed to modulate dream experience, influencing it by brain stimulation techniques (e.g., [26]).

Considering this background, here, we intend to summarize the available findings on the neural basis of dreaming with a focus on the most recent studies supporting the two major hypotheses about the production, elaboration and recall of dream experience. Further, we provide a comprehensive update about the findings resulting from stimulation protocols aimed to manipulate sleep mentation. Finally, based on the available empirical evidence, we intend to propose promising directions for future studies on dream experience.

## 2. Overview of Current Knowledge

The neuroscientific study of dreaming has undergone remarkable advances thanks to different techniques of investigation: (a) Electroencephalographic (EEG) and polysomnographic (PSG) protocols, or video-PSG recordings with provoked awakenings; (b) intra-cranial/stereo-EEG (iEEG); (c) functional neuroimaging, such as Positron-Emission Tomography (PET), Functional Magnetic Resonance Imaging (fMRI); (d) structural neuroimaging, such as MRI and MRI associated with Diffusion Tensor Imaging (DTI).

### 2.1. Continuity Hypothesis

The original “continuity hypothesis”, proposed in the early 1970s [27], posited for the first time that personal concerns and conceptions characterizing the waking thoughts have their own continuity in sleep [28]. Dream experts broadly discussed this assumption, and many investigations were aimed to test it, considering different aspects of mental activity during sleep. Several re-interpretation and adjustments were made to the continuity hypothesis. Indeed, many researchers concluded that dreams reflect the waking-life and stated that dream contents could represent the incorporation of daily experience. In particular, studies suggested that the most emotionally intense waking events were incorporated during mental sleep activity [29]. More directly, the incorporated memories of waking events occurring 1–2 days before the dream were defined as “day-residue effect”, while life experiences related to 5–7 days before the dream were called “dream-lag effect [30]. This phenomenon was mainly found in association with Rapid-Eye Movement (REM) sleep dreams [31]. In this respect, some authors hypothesized that the dream-lag effect might represent the transfer of new memory representations from the hippocampus to the neocortical circuits, providing a gradual integration into older representation [32]. Specifically, the dream-lag effect could be the expression of emotional memory processing during REM sleep [23,33].

This evidence assumes that dreaming and memory processes are interrelated. In this view, some studies started from the idea that dream recall (DR) upon awakening could be considered a peculiar form of declarative memory and, more specifically, of episodic memory [3,5,8]. PSG with provoked awakenings is the gold standard method to study DR [34]. At the beginning of the 21st century, several studies started to investigate EEG activity preceding the DR to identify specific patterns of activity that could predict the presence of dream experience [2,3,4,5]. Convergent findings pointed to the relationship between frontal theta oscillations (5–7 Hz) and DR upon awakening from REM sleep [5,8,10]. Moreover, the frontal theta activity during REM sleep seems to be a predictor of recent waking-life experiences [29]. Additionally, a decreased alpha activity (8–12 Hz) over the parieto-occipital region could be a predictor of DR from stage 2 non-REM (NREM) sleep [3,5]. Partly in line with this pattern, Takeuchi et al. [2] found that recall condition was predicted by reduced alpha and sigma power in central areas during NREM sleep onset periods. Nevertheless, they also revealed that higher alpha and sigma (12–15 Hz) power in the same regions were related to DR during REM sleep onset [2].

It should be considered that both alpha and theta activity are linked to mnestic neural processes during wakefulness [35]. Specifically, increased frontal theta oscillations and reduced parieto-occipital alpha activity predict good performances in episodic memory tasks during the waking state [36]. Consistently, an iEEG study on patients with drug-resistant epilepsy showed that DR was associated with the medial temporal lobe activity, which is strongly implicated in memory processes [37]. Specifically, the authors found that high recallers showed enhanced rhinal-hippocampal and intrahippocampal EEG coherence in all frequency bands (especially in the theta band), underlining that anatomic and functional connectivity between limbic structures was crucial both for waking declarative memories and DR [37].

In light of this, the continuity hypothesis could be extended to a neurobiological perspective. In fact, these patterns are responsible for the encoding and retrieving episodic memories in both sleep and waking-life, indicating the existence of shared mechanisms for cognitive elaboration across different states of consciousness [38].

In this regard, neuroimaging studies found that specific structures involved in cognitive and emotional processes during wakefulness are activated during sleep. It should be noted that most authors focused on REM sleep, according to the traditional belief that dreaming is an epiphenomenon of this sleep stage [39]. Specifically, studies by means of PET and fMRI highlighted some REM sleep neural correlates overlapping to those of dreaming. These functional neuroimaging studies demonstrated that limbic and paralimbic structures, thalamus, basal forebrain, pontine tegmentum are significantly activated during REM Sleep [40,41,42].

It is well-established that amygdaloid complexes, hippocampal formation and anterior cingulate cortex have a pivotal role in emotional memory encoding and consolidation. During REM sleep, their activation may explain the emotional load (i.e., emotional intensity) of some dream contents [40,41,42,43]. Although this sleep stage is characterized by muscle atonia [44], neuroimaging studies found higher activation over motor and premotor regions, consistently with the fact that dreamers often are engaged in motor behaviors [40,45,46].

Conversely, other regions appear to be hypoactivated compared with the wakefulness, such as the precuneus, orbitofrontal cortex, posterior cingulate gyrus and dorsolateral prefrontal cortex [41,42,43]. The reduced activation in these areas is related to the deficit in executive function, time perception and insight during dreaming [43].

Most of the mentioned findings stem from protocols that did not actually collect dream reports after awakening, based on an ideal correspondence between REM sleep and dream generation. Structural brain imaging studies overcame this assumption and focus on morphoanatomical parameters (e.g., volumetric measures) and interindividual variability of dream features.

For example, DTI is an MRI technique that examines the white matter integrity using anisotropic diffusion to estimate the neuroanatomical organization of the brain [47]. Employing this approach De Gennaro et al. [6] found that volume and diffusivity (magnitude of neuronal water diffusion) of the hippocampus and amygdala correlated with qualitative dream features. Specifically, left amygdala volume was related to the bizarreness of dreams, while a smaller volume of the left hippocampus and a larger volume of the right hippocampus was linked to emotional load. Additionally, the lower structural integrity of the left amygdala was associated both with reduced emotional load and shorter dream reports [9]. More recently, an MRI study on patients affected by Parkinson’s disease (PD) replicated these findings [15]. Measures of subcortical volumes and cortical thickness also revealed that visual vividness is related to the amygdalae and the thickness of the left medial prefrontal cortex (mPFC). In addition, the emotional intensity was positively correlated with hippocampal volume. PD patients under pharmacological treatment can also be considered a model to study the dopaminergic system involvement in dreaming. In other words, a higher dosage of dopamine agonists indicated a hypodopaminergic state. Authors reported that dopamine dosage negatively correlates with dream bizarreness and emotional load in patients with PD [15]. On the one hand, these findings confirmed that the mesocortical–mesolimbic dopamine system has a crucial role in dream phenomenology, as suggested by Solms’ observations [19]. On the other hand, the evidence of the mPFC and dopamine level contribution in qualitative aspects of dreaming supports the neurophysiological continuity-hypothesis, given the well-established role of the mesolimbic-dopaminergic system in modulating reward and motivational behaviors [48].

Interestingly, these findings pointed to the existence of specific stable characteristics of brain structures impacting on the qualitative features of dream reports.

In keeping with these studies, another research group tested the interindividual differences between people with higher dream recall rate (High Recallers, HR) and low recall rate (Low Recallers, LR). Differences between HR and LR were found both during wakefulness and sleep [7,49,50]. Eichenlaub et al. [7,49] using PET highlighted that HR increased cerebral blood flow than LR in the mPFC and temporo-parietal junction (TPJ) during REM sleep, N3 and wakefulness [49]. More recently, the authors evaluated the association between brain anatomical structures and dream recall rate, focusing on mPFC, TPJ and limbic structures (amygdala and hippocampus) [51]. They showed significant differences between groups in the white matter of the mPFC. In addition, the authors confirmed no differences in the amygdala and hippocampus concerning dream frequency, confirming previous results [6,15].

Overall, the findings suggest that amygdala and hippocampus have a parallel role in processing emotional memories during both wakefulness and sleep. The involvement of these subcortical nuclei in dream affect has also been confirmed by two recent studies on brain-damaged patients [52,53]. Specifically, the analysis of dreaming collected from subjects with bilateral amygdala lesions showed that patients had more pleasant and significantly shorter dream reports than control subjects [52]. Accordingly, these patients also had impaired emotional episodic memory during waking state [52]. Another study on subjects with bilateral hippocampal damage found that patients had lower DR frequency and less episodic-like contents [53].

Moreover, converging results provide evidence in favor of the role of mPFC in dream production and recall [19]. The frontal EEG theta power associated with DR [5,8,10,34] can be the expression of the involvement of mPFC in the oneiric activity.

It should be noted that the mPFC, along with the TPJ, is part of the Default Mode Network (DMN), the neural circuits underlying resting state, internally oriented mental processes (e.g., mental imagery, mind-wandering) and episodic memory retrieval [43,54,55]. Both mind-wandering (or daydreaming) and dreaming include thoughts, sensations, visual-imaginative elements and are characterized by a relative lack of meta-awareness [56]. According to the authors hypothesized that dreaming is an intensified expression of daydreaming [54], we point out that these phenomena share the same neural correlates suggesting, once again, a certain continuity between physiological/neurobiological mechanisms subserving cognitive processes in sleep and wakefulness.

Based on this view, some authors combined fMRI with machine learning models with the aim to assess the similarity between waking visual experiences and dreaming including visual elements [57]. They applied decoding models trained on stimulus-induced brain activity in visual areas during wakefulness and showed an accurate categorization and detection of mental contents. Specifically, they used statistical decoders trained to predict categorical labels of viewed objects/scenes. The target categories to be decoded from brain activity were constructed from dream contents consisted of 20 object categories for each subject. The results revealed that objects or scenes visualized during mental sleep activity could be predicted from specific brain activity patterns. These findings suggested that visual dream contents are represented by discriminative brain activity patterns similar to perception in the visual cortex [57].

In addition, the relation between waking visual experience and dreaming was showed from previous iEEG study across the medial temporal lobe and neocortex during sleep, wakefulness and visual stimulation with fixation [58]. By using 72 depth electrodes in neurosurgical epilepsy patients, the authors had the great advantage to provide anatomically and temporally accurate information about the relation between rapid-eye movements (REMs) and underlying activity in visual regions. In fact, REMs onsets are associated, during sleep and waking state, with specific intracranial potentials that resemble ponte-geniculate-occipital waves [58]. This study reported that single neurons in the medial temporal lobe showed lower firing rates prior to REMs and increasing rates immediately after REMs, similarly to the pattern upon image presentation during fixation in waking state. More directly, the “scanning hypothesis” suggests that REMs during sleep imitate the scanning of dream scenes [59]. Consistently, a study on patients with REM Behavior Disorder, RBD (i.e., a sleep disorder characterized by intermittent loss of normal muscle atonia REM sleep with the appearance of complex motor behaviors associated with dream mentation) found a substantial concordance between the directions of REMs and the dream scenario with goal-oriented action during RBD [59]. Consistently, a recent study by Laberge et al. [60] provided an argument that visual imagery during REM sleep is similar to visual perception during wakefulness. The authors examined smooth pursuit eye movements during tracking of a slow-moving visual target during REM lucid dreaming and showed highly similar smooth pursuit tracking during both waking perception and REM sleep [60].

Overall, this “brain reading” approach suggests that specific visual experiences during sleep are predicted by the same brain patterns of stimulus perception. In other words, the results support the hypothesis of common neural substrates for waking perception, imagery and visual dream experience. However, it should be noted that the observation by Horikawa et al. [57] was limited to the wake–sleep transition, and this implies that they studied a dream-like state characterized by hypnagogic hallucinations.

### 2.2. Activation Hypothesis

Besides the idea that different states of consciousness share similar neurobiological mechanisms, many findings converge to the hypothesis that also the brain activation level during sleep promotes dream recall (DR). Actually, Koulack and Goodenough by their “Arousal-retrieval Model”, posited that the mental activity during sleep could be memorized only when sleep becomes fragmented and short awakenings occur, allowing the dream material to be elaborated and transferred from short-term memory storage to long-term storage [61].

Hobson and McCarley [62], shortly after, proposed a neurophysiological model of dreaming: the Activation-Synthesis hypothesis. The model started by assuming that there is an isomorphism between simultaneous physiological and psychological events during sleep mentation. They postulated that dreaming resulted from the periodic activation of the forebrain during sleep. The authors also introduced for the first time the idea that EEG desynchronization may be involved in oneiric processes [62]. However, it should be noted that all these considerations were based on REM-dreaming equivalence.

In the 1990s, Antrobus introduced the “Activation Model”, suggesting a link between dreaming and local periodic activations during sleep of the same cerebral structures designated for perception, motor and cognitive elaboration during wakefulness [63].

In keeping with this background, more recently, some studies found that DR was hampered when sleep was characterized by a great amount of slow wave sleep (SWS) and lower awakenings (e.g., [64]). Subjects with high rates of DR showed increased intra-sleep wakefulness [50,65]. In addition, sleep fragmentation in older people was also related to qualitative aspects of DR. Indeed, the wakefulness after sleep onset during undisturbed night was correlated with high visual vividness of dream reports [18].

Along the same vein, subjects did not recall dream experience during a recovery night after sleep deprivation. De Gennaro et al. [64] showed that the greater proportion of SWS—characterized by higher slow wave activity (SWA; 0.5/1–4 Hz)—and the reduced percentage of REMs during recovery night were related with a remarkable difficulty in DR after morning awakening. This could represent a consequence of the elaboration of reduced “dreamlike” materials over the night. It should be noted that SWA decreases across the night as a function of homeostatic sleep pressure and this could explain why dreams are more likely recalled in the early morning hours, sometimes making NREM sleep dream reports indistinguishable from REM sleep dream reports [66,67].

More directly, EEG investigations revealed the link between specific topographic patterns and dream experience [4,9,11,12,13,14,18]. Overall, the EEG findings point out that the local presence of slow waves in certain regions may interfere with the encoding and recall of dream experience. Zhang et al. [11] reported that lower global slow oscillations (<1 Hz) were associated with the successful retrieval of dream contents. Additionally, DR would appear when the sleep EEG during NREM sleep was characterized by lower SWA (0.5/1–4 Hz) in the centro-parietal area [4], in the left frontal and temporo-parietal regions [9], and in the parietal region [18]. Siclari et al. [13] found that dream experience was related to reduced SWA over parieto-occipital regions both during REM and NREM sleep. In parallel, the brain activation expressed in term of higher rapid frequencies (>25 Hz) predicted DR when localized in the parieto-occipital areas during NREM sleep and in frontal and temporal regions during REM sleep [13]. Similarly, higher beta power (16–24 Hz) is related to DR after REM sleep over the occipital area in a multiple awakenings protocol [12]. However, it should be mentioned that a recent investigation by Wong et al. [68] using a serial awakening paradigm failed to find any difference between the recall and non-recall condition.

Interestingly, investigations on clinical samples support the activation-hypothesis. Patients with insomnia disorder reported greater DR frequency [69]. Consistently with the Arousal-retrieval Model [61] the relation between insomnia and DR rate may be explained by the amount of awakenings during the night [69]. Moreover, it should be considered that insomnia patients showed a reduced delta/beta ratio, which had proved to be reliable index of brain activation [70,71]. Although no direct studies have been carried out on EEG correlates of DR in insomniacs, it may be hypothesized that the presence of higher proportion of fast-frequency EEG activity could facilitate DR in these patients. Additionally, a recent study on the neural correlates of dreaming in narcoleptics partly confirmed the role of EEG desynchronization in REM and NREM sleep in promoting DR [16]. D’Atri et al. [16] found that recall condition was associated with lower values of delta/beta ratio compared with non-recall. In particular, lower delta power in centro-parietal areas during both sleep stages, and higher beta power in the same regions during NREM predicted DR [16]. Similarly, narcoleptic subjects reporting lucid dreams (i.e., the experience of being aware of dreaming during sleep) showed lower delta activity during REM sleep than individuals without lucid dreams [72]. This finding suggests that a more activated brain may represent a privileged context to promote cognitive processing.

Overall, many studies identified the activation over posterior and anterior regions, confirming, again, that they are involved in DR, consistent with earlier studies [19].

## 3. Manipulating Dream Experience

### 3.1. Sensory Stimulation and Dreaming

Thus far, we have looked at the available studies that made an effort in order to understand the neural correlates of sleep mentation. However, it should be mentioned that, in parallel, multiple older investigations were aimed to directly influence the ongoing dream scenario by using different kind of stimulations, i.e., visual, auditory, somatosensory or olfactory cues.

The first attempt to manipulate dreaming with pre-sleeping external stimuli was reported by Dement and Wolpert [73]. The authors deprived subjects of fluids one day prior to sleeping and obtained 5 out of 15 REM dreams, including thirst-related content. Goodenough et al. [74] used stressful films during a pre-sleep period, demonstrating that visual stimulus can increase dreams characterized by negative emotional tone. Moreover, subjects exposed to visual inverting prisms recalled more vivid dreams [75].

Visual stimuli were also applied during sleep. Rechtschaffen and Foulkes [76] presented some images during REM Sleep, while subjects’ eyes were taped open. Nevertheless, no incorporation was found.

Concerning auditory stimuli, Berger [77] found that presenting personally significant names during REM sleep provoked a high rate of indirect incorporation of these stimuli into dream experience. For instance, the names were modified for assonance or in an associative manner [77]. Similarly, Hoelscher et al. [78] revealed that words with a relevant personal meaning were more frequently incorporated than other words.

Visual and auditory modalities were combined in a study that applied stimulation both during REM and stage 2 NREM sleep [79]. Interestingly, the effect was found only when the stimulation occurring in NREM sleep, after which people reported dream with more visual contents [79]. Although the authors suggested that REM sleep was not very sensitive to visual stimuli, some researchers investigated lucid dreaming, positing that visual stimuli could be incorporated into the dream experience [80]. Indeed, a study in which participants were requested to wear a LED light-fitted mask detecting rapid eye movements and sending visual stimuli showed that sleeping subjects realized that they were currently in REM sleep stage, increasing the possibility of producing lucid dreams. Participants frequently reported a dream scenario characterized by illuminated environments or containing street lamps [80].

It should be noted that researchers also shaped dreaming by introducing specific bodily stimulus during sleep. For instance, a spray of water on the subjects’ skin was applied [73], as well as a thermal stimulation [81,82]. Specifically, Dement and Wolpert by using various external stimuli (i.e., 1000-cps tone stimulus, light flashes, water spray and arousing bell) did not show remarkable effects in modifying the dream content, nevertheless cold water appeared more effective than other stimuli [73]. Some authors also tried to stimulate subjects with vestibular stimulation, for instance asking subjects to sleep in hammocks [83]. Other studies evaluated the effect of tactile stimulation by a pressure cuff to a leg, both during REM [84,85] and at sleep onset (stage 1 NREM sleep; [86]) provoking high incorporation rates, with a percentage of 40%–80%. Furthermore, the incorporation of somatosensory stimuli was obtained by electrical pulses on the wrist to stimulate muscle contractions during REM sleep [87]. Overall, these kinds of stimulations increased vividness and higher bodily sensations in the oneiric contents.

More recently, olfactory stimulation was successfully used to modulate dreams’ emotional tone during REM sleep [88]. Indeed, the authors showed that pleasant scent (e.g., rose) were associated with positive dream experience, while unpleasant smell (e.g., eggs) with more negative dream experiences [88]. Furthermore, it was demonstrated that using an odor presented during REM sleep and previously associated with an image during waking state induced subjects to report a dream experience containing the related image [89].

The findings of the impact of external stimuli on dream contents are mixed, and no compelling explanation about these phenomena was reported. In fact, the results on dream incorporation are discordant between studies and/or stimuli often fail to be incorporated in the content of dreams directly [1,76,77]. Moreover, the method used to classify the incorporation is quite different among studies. The judgment of whether a stimulus had been actually represented into the dream scenario or not was often subjective (e.g., [73]) and only few studies tried to assess, by an objective method, the presence/absence of incorporation and themes/emotional contents of dreams by identifying defined categories and requiring rating to two independents judges (e.g., [77,89]). However, a systematic and reliable method to study the incorporation is still lacking.

To some extent, these studies showed that the sleeping brain is able to perceive and process much information integrating them in an oneiric narrative. However, some authors suggested that stimuli incorporation into dreams cause a sort of micro-arousals that slightly awaken the sleeping subject so that they perceive it, but not enough to wake them up [90]. This view seems to be relatively compatible with the activation-hypothesis, however only scarce evidence about neural correlates is available.

### 3.2. Brain Stimulation and Dreaming

A very innovative line of research is aimed to manipulate dreaming by modulating brain activity directly. This method is based on non-invasive brain stimulation techniques and may be considered complementary to the earlier mentioned protocols influencing dream contents.

Specifically, Transcranial Direct Current Stimulation (tDCS) can induce focal changes in cortical excitability by a constant low-intensity current [91]. It is well-established that during wakefulness tDCS can alter the activation of motor [92], somatosensory [93], prefrontal [94] and visual cortices [95]. Additionally, tDCS can impact on cognitive functions, namely working memory [96] or tactile perception [97]. In recent years, a growing number of studies have investigated the effects of these techniques in the context of sleep and vigilance [98,99,100,101,102,103].

Starting from this knowledge, some investigations assessed tDCS effects on dreaming, intending to explore the involvement of different cortical areas in the oneiric experience. In a pioneering study, the stimulation was applied during Stage 2 NREM sleep over frontal (cathodal) and right posterior parietal (anodal) cortex [104]. The authors found a greater number of visual imagery reports after tDCS than sham condition, but the same was revealed for the two control conditions (i.e., reversed polarity and other-cephalic tDCS). Hence, the effects seem to be independent of tDCS polarity and the higher visual dream frequency appears to be elicited by a general arousing effect, rather than depending on the specific stimulation of cortical areas [104]. Moreover, no effect was observed during REM sleep or SWS [105,106]

Bihemispheric tDCS has been recently applied during REM sleep to investigate the potential interference of tDCS over the sensorimotor cortex with movement and bodily sensations of dream experience [107]. The study showed that subjects awakening after stimulation had a lower rate of reports including movements (especially repetitive actions), while tactile and vestibular sensations were not affected by tDCS. Hence, the authors suggested that sensorimotor cortex is responsible for generating dream movement, confirming that the neural bases of peculiar dream contents are shared between sleep and waking state.

The absence of awareness, and the lack of voluntary control during dream experience are both intriguing issues for dream scientists. In this regard, the researchers started becoming interested in modulating the level of consciousness during sleep, namely, inducing lucid dream experiences [26,108,109]. Hobson et al. [110] proposed that lucid dreaming may stem from the reactivation of dorsolateral prefrontal cortex that, as mentioned, appear deactivated during REM Sleep [41,42,43]. In this vein, Stumbrys et al. [108] applied tDCS stimulation over the dorsolateral prefrontal cortex during REM sleep and observed a small increase of self-reported lucid dreaming only in frequent lucid dreamers.

Further, transcranial alternating current stimulation (tACS) was applied to induce dream lucidity [26]. tACS exerts its effects by the application of an alternating current to the scalp at a specific frequency. The alternating stimulation drives the cortical network to oscillate at the given frequency, inducing periodic shifts in the transmembrane potential of underlying neurons [111]. Bearing in mind the involvement of frontal areas in dream activity [19], Voss et al. [26] used tACS for 30 s at several different frequencies over fronto-lateral regions during REM sleep. Subjects reported lucid dreams when 25 and 40 Hz stimulation were applied. Conversely, a more recent study on lucid dreaming did not support this evidence, observing no effect of 40 Hz frontal stimulation [109].

To our knowledge, the only one study using Transcranial Magnetic Stimulation (TMS) tried to manipulate dream experience targeting the posterior parietal cortex. TMS was applied during NREM sleep before awakening subjects and asking them about the presence/absence of dream experience [112]. The results showed that TMS induces an EEG response characterized by a larger negative deflection (similar to a larger NREM sleep slow wave) and a shorter period of phase-locking associated with non-recall compared to recall conditions [112]. Additionally, the amplitude of this deflection was negatively correlated with the total word count of dream reports. This response is typical of cortical circuits in condition of bistability between depolarized up-states and hyperpolarized down-states [113]. Notably, changes in the bistability of cerebral networks, mainly expressed in the form of slow oscillations, modulate the level of consciousness and the ability of the cortex to integrate information [112]. The authors started from the view that dreaming represents a consciousness experience during sleep. In turn, the level of consciousness corresponds to the amount of integrated information generated by a complex of elements and would only vanish during dreamless sleep or under general anesthesia [114]. In other words, slow frequencies (1–4 Hz) EEG during sleep are associated with neuronal down-states preventing the emergence of stable causal interactions among cortical areas and provoking the loss of consciousness [115,116,117].

Although the application of brain stimulation techniques in the field of sleep and dreaming represents a promising research area [118], the available evidence is far from being conclusive.

However, the direct manipulation of brain activity could give us powerful insights in understanding the neural substrates of dream recall.

### 3.3. Insights for Future Research

Dream research using EEG and neuroimaging techniques have advanced significantly over the last twenty years. However, several issues are still opened and unsolved. To direct future studies, we tried to summarize the questions deserving further deepening.

Firstly, we have to underline that, to date, different techniques allowed researchers to assess just some pieces of knowledge on dream experience. We highlighted that some studies aiming to detect structural and functional measures or brain activation patterns revealed exclusively stable/trait-like characteristics of subjects who recall their dreams (e.g., [6,7,15,49]).

Many researchers agree to consider PSG/EEG studies with provoked awakenings the gold-standard method to study dream recall (DR), since it is a tool easy to apply. On one hand, PSG allows to carry out very different experimental protocols (nap, multiple naps, multiple awakenings, multiple sleep latency test, undisturbed night). On the other hand, these have often led to conflicting or partially consistent findings.

In this respect, we point that most investigations tried to find the possible relations between electrical activity patterns and DR without considering a crucial issue: are these patterns state-like or trait-like phenomena? In this respect, EEG within-subject protocols (e.g., [8,9,13,16]) would ensure that the observed patterns depend on the specific physiological background and not the stable EEG characteristics of recorded subjects. However, no data about how these aspects impact on dream contents are still available.

Further, very few studies tried to combine different methods to detect the neurobiological correlates of dreaming (e.g., [49]). We also need to consider that variation in dream experience can be due to homeostatic and circadian factors which can play a role in the specific scenario of physiological activity during sleep [119]. For instance, Goodenough et al. [120] found dream reports with higher total word count followed NREM sleep, in the process of transitioning to REM [120]. To date, just a few studies have investigated the influence of circadian and homeostatic aspects on dreaming [4,9,12,16] showing, overall, that they do not affect the EEG pattern related to the presence/absence of DR. However, none of these studies collected dream reports, so no information is available about the relation between the EEG variations, due to these processes, and the qualitative/quantitative features of DR.

Multiple awakenings and within-subject studies can assess both state- and trait-like variables and their interactions with homeostatic pressure and chronobiological variables (e.g., ultradian oscillation, a switch-like circadian oscillation, or 28-day cicatrigintan rhythm for women, [119]). However, the use of the serial awakening method to investigate the EEG correlates of dreaming has been criticized (e.g., [121]) since this paradigm has not always produced consistent results [68]. We believe that complementary studies with a focus on undisturbed sleep and neuroimaging studies can help put the pieces of this complex puzzle together. More directly, future studies should be taken into consideration the possibility of integrating different techniques to solve the state-/trait-like issue and assess the role of brain networks involved in dreaming at multiple levels (structural measures, activation patterns, EEG activity in cortical and subcortical regions).

We highlighted that two main models (activation and continuity) may explain the production and retrieval of dream experience. Overall, in line with the earlier neuropsychological findings, more recent neuroimaging and EEG data confirmed that specific brain networks drive many aspects of dream experience. The current literature suggested that the local activation of the posterior zone and medial prefrontal cortex (mPFC) are involved with dream cognition [15,51]. Hence, the neuroscientific discoveries are in line with the continuity hypothesis since they mostly converge to show that the same brain networks responsible for cognitive functions during wakefulness play a role in dream experience. Additionally, specific EEG oscillations underlying memory processing such as theta and alpha bands have proven to predict DR (e.g., [5]). In parallel, the presence of a significant level of arousal expressed in terms of higher rapid EEG frequency (beta, gamma range) and/or lower slow EEG frequency (e.g., [9,13,16]) seem to conflict with the idea of “continuity” between sleep and wakefulness. Notwithstanding, the models based on physiological activation and shared mechanisms across the different state of consciousness are not mutually exclusive: both provide a better comprehension of the neural correlates of a complex phenomenon as dreaming.

It should be speculated that the continuity hypothesis is mostly related to the mechanism involved to recall specific memories (autobiographical and/or emotionally charged memories) inserted to some extent in dream activity. At the same time, the brain activation during sleep would produce a physiological background promoting the storage of oneiric-mnestic traces. In keeping with the idea that the two models are complementary, Koulack and Goodenough [61] suggested that only salient events or particularly emotional intense contents that stand out compared to other stimuli are processed at the time of arousal and are more likely recalled after awakening.

Here, we have also mentioned that dream contents and the level of consciousness during sleep may be manipulated. The few studies investigating induction of lucid dreams with electrical brain stimulation (tDCS and tACS) have found fascinating but sparse effects on dreaming [26,107,108,109], and a method for reliably inducing lucid dreams by electrical stimulation of the brain is still yet to be found. Moreover, further applications of TMS should be expanded to change the level of consciousness [112]. For instance, high-frequency repetitive TMS (rTMS) may be applied to enhance neuronal excitability in focal cortical regions inducing specific EEG frequency to produce lucid dreaming [122].

In our opinion, the application of brain stimulation to modulate the level of consciousness and induce specific dream contents may give us crucial insights on the neural correlates of dreaming. In this vein, future studies may plan to stimulate different brain areas by using different types of stimulation.

More in general, both results from sensory and brain stimulation are in line with the idea that the neural bases of specific oneiric contents coincide with those related to corresponding waking behavioral and cognitive processes. Undoubtedly, the study of incorporation is still challenging for researchers mostly because of the intrinsic access limitation to dream that cannot be investigated directly.

A novel approach to achieve a better understanding of this phenomenon is represented by the study on parasomnia-like events. Many findings pinpoint that the content of dream enacting behaviors (DEB) had a high level of concordance with subsequent dream reports [123]. In this respect, several studies agree on the idea that REM Behavior Disorders (RBD) or sleep talking could be considered a viable way to access to dream contents [123,124]. Moreover, some pioneering studies by the group of Isabelle Arnulf focused on sleepwalking and sleep terrors [125,126]. It should be noted that the uniqueness of these studies is represented by the dissociation between cortical areal showing slow-waves and motor cortices showing a wake-like activation [127]. In such way, this approach may provide useful insights on the dream characteristics associated to SWA and, more in general, to deep sleep.

Hence, we think that the investigation on parasomnia is promising future directions in dream research since the behavioral enactment (e.g., movements, verbalizations, emotional reactions) of dream scenario would allow researchers to look at dream contents while the subject is still asleep directly.

Table 1 illustrates the mentioned neuroscientific techniques, with their strengths and weaknesses, highlighting that each of these approaches led researchers to identify specific aspects of neural correlates of dreaming and solve different dreaming-related issues.

### 3.4. Conclusions

We report in Table 2 a short synopsis of the studies that have been discussed in this review as a function of the level of investigation (i.e., EEG, iEEG, etc.) and of the support to a continuity or activation hypothesis.

To sum up the points in a future research agenda, we emphasize that the following issues should be included:

1. Studies aimed to collect dream reports using within-subjects design protocols, taking under control the homeostatic and circadian variables, should be planned.
2. Protocols collecting dream recall (DR) with high-density EEG (high spatial resolution) should be implemented to replicate previous findings on the relationship between specific oscillations and qualitative dream features. Source localization technique should be integrated to maximize the matching between cortical regions and dream contents.
3. Combining different techniques would benefit the dream research, also integrating brain stimulation techniques (tDCS, tACS and TMS).
4. Providing longitudinal data in clinical and healthy samples to observe potential changes in the brain patterns and dream contents may help face the state-/trait-like issue.
5. Studies with a focus on parasomnia-like events would allow overcoming the problem of correspondence between specific time/stage of sleep and the DR generation.

Finally, it should be considered that collaboration among the scientists in the relatively wide dream community could reduce the high fragmentation of the current literature, providing, for instance, a more unified method to study dream experience.

## Figures and Tables

**Table 1:** Neuroscience techniques for the study of dreaming.

Technique	Definition	Strengths	Weaknesses	Dreaming-Related Issues
**iEEG**	An invasive technique that uses electrodes placed directly on the exposed surface of the brain to record electrical activity from the cerebral cortex.	iEEG signal provides anatomically accurate information about the involvement of neuronal populations and precise temporal information iEEG is a useful tool for decoding neural computations and interregional connectivity iEEG allows to study the electrical activity in subcortical nuclei	Invasive tool Limited Accessibility (clinical setting) Limited to small group of subjects (patients with epilepsy)	Studies of subcortical regions involved in emotional and memory processes Potential coupling of dream mentation while recording cerebral activity
**PSG or VPSG**	A non-invasive measure used to study sleep, also as a diagnostic tool. PGS allows a continuous recording of specific physiological variables combining a different kind of electrodes or sensors: - EEG (or hd-EEG) records the electrical activity across the scalp produced by neural activity in underlying brain regions - EOG is applied to monitor eye movements - EMG is applied to monitor the muscle tone Additionally, breathing, leg movements, and heart rate can be recorded. VPSG combines simultaneous PSG and video-recording to evaluate patients with nocturnal events.	Easy montage suited to implement different protocols PSG had high temporal resolution; PSG with hd-EEG allows to enhance the spatial resolution PSG can be easily combined with provoked awakenings PSG allows to carry out multiple awakenings from different sleep stages and circadian windows PSG led to carried out more easily within-subjects design (same subject recorded more than once) PSG can be combined with brain stimulation techniques VPSG is used in patients with nocturnal behavior/parasomnia and allow to correlate behavior with neurophysiologic parameters	Low spatial resolution Skin redness at site of electrodes placement Scalp Electrodes can cause some discomfort during the night recording	PSG allows to determine possible causal links between targeted brain areas and cognitive dream-related processes Overcome the equation REM-Dreaming Understanding the influence of homeostatic and circadian processes State-/Trait-like issue Investigation of DEB as direct access to dreaming
**fMRI**	A non-invasive technique that measures the changes in cerebral blood flow associated with neural activity. fMRI allows the assessment of the normal functional anatomy of the brain.	High spatial resolution to detect the activation of specific neural circuits fMRI studies can be extended to subcortical nuclei Potential EEG-fMRI combination	Low temporal resolution Complex equipment Extensive costs Presence of system noise that potentially affects continuity of sleep Intrinsic limitations in collecting measures contingent to dream experience	Understanding of the relationships between specific dream features and brain activity Studies of brain regions involved in emotional processes during sleep
**PET**	A non-invasive technique to obtain structural image of the brain measuring levels of glucose to detect active neurons.	High spatial resolution to detect the activation of specific neural circuits PET studies can be extended to subcortical nuclei Potential EEG-fMRI combination	Low temporal resolution Complex equipment Radioactive tracer is injected into participants Extensive costs Intrinsic limitations in collecting measures contingent to dream experience	Understanding of the relationships between specific dream features and brain activity Studies of brain regions involved in emotional processes during sleep
**MRI (and DTI-MRI)**	A non-invasive technique that provides the measure of structures of the brain. DTI-MRI allows examining the white matter tracts using anisotropic diffusion to estimate the neuroanatomical organization of the brain.	Examination of neuroanatomical parameters MRI-DTI studies can be extended to subcortical nuclei	Low temporal resolution Complex equipment Extensive costs Assumes that differences in dream recall are related to basic morfo-anatomical differences	Structural/volumetric measures can provide a reliable and stable measure to account for some trait-like variables of dream experience (State/Trait-Like issue) Studies on the role of subcortical structures
**tES (tDCS and tACS)**	A non-invasive brain stimulation technique that passes an electrical (direct, tDCS or alternating, tACS) current through the cortex in to modulate brain function.	Relatively easy and non -invasive tool Portable equipment tES can be easily combined with PSG/EEG/hd-EEG	Noise or tactile sensations can provoke awakenings or micro-arousals Limited spatial accuracy Many variables to check for an optimal stimulation protocol	Manipulating Dreams Inducing Lucid Dreams
**TMS (or rTMS)**	A non-invasive brain stimulation technique in which a changing magnetic field is applied to increase neuronal excitability in specific area.	TMS can be easily combined with PSG/EEG/hd-EEG Focality of stimulation (vs. tES)	Noise or tactile sensations can provoke awakenings or micro-arousals Skin redness at site of coil placement	Manipulating Dreams Inducing Lucid Dreams

iEEG, Intracranial-Electroencephalography; PSG, Polysomnography; VPSG, video-polysomnography; EEG, Electroencephalography; hd-EEG, High-Density Electroencephalography; EOG, Electrooculography; EMG, Electromyography; DEB, Dream Enacting Behavior; fMRI; Functional Magnetic Resonance Imaging; PET, Positron-Emission Tomography; MRI; DTI, Diffusion Tensor Imaging; DR, Dream Recall; tES, Transcranial Electrical Stimulation; tDCS, Transcranial Direct Current Stimulation, tACS, Transcranial Alternating Current Stimulation; TMS, Transcranial Magnetic Stimulation, rTMS, Repetitive Transcranial Magnetic Stimulation.

**Table 2:** Main findings on neurobiological mechanisms of dreaming.

Approach	Main Patterns Related to Dreaming	Continuity	Activation
**EEG/PSG**	Higher frontal theta oscillations (5–7 Hz) during REM sleep [5,8,10]	**X**	
	Lower parieto-occipital alpha power (8–12 Hz) during 2NREM sleep [3,5]	**X**	
	Lower SWA (0.5/1–4 Hz) in the centro-parietal area [4], in left frontal and temporo-parietal regions [9], and in the parietal region [13,18] during 2NREM sleep Lower SWA in parieto-occipital regions during REM sleep [13] Lower delta power in centro-parietal areas during REM and NREM sleep in narcoleptic subjects [16]		**X**
	Higher rapid frequencies (>25 Hz) in the parieto-occipital areas during NREM sleep and in frontal and temporal regions during REM sleep [13] Higher beta power (16–24 Hz) in the occipital area during REM sleep [12] Higher beta power in centro-parietal areas during NREM sleep in narcoleptic subjects [16]		**X**
	Higher intra-sleep wakefulness in high recallers [49,65] Higher intra-sleep wakefulness correlates with high visual vividness [18]		**X**
**iEEG**	Higher rhinal-hippocampal and intrahippocampal EEG coherence in all frequency bands (especially in the theta band) in high recallers [37]	**X**	
	Lower firing rates prior to REMs and increasing rates immediately after REMs in single neurons of the medial temporal lobe [58]	**X**	
**Neuropsychological**	Lesions in the right and unilateral posterior networks alter dream features [19] Lesions in the bilateral anterior system causes dream cessation [19] Prefrontal leucotomy and lesions in the medial prefrontal cortex cause dream cessation and excesses of dreaming, respectively [19]	**X**	
	Bilateral amygdala lesions alter dream features (more pleasant and shorter DR) [52] Bilateral hippocampal lesions cause lower DR frequency and less episodic-like contents [53]	**X**	
**Functional neuroimaging**	Activation of limbic and paralimbic structures, thalamus, basal forebrain, pontine tegmentum during REM sleep [40,41,42]	**X**	
	Increased cerebral blood flow in high recallers over the medial prefrontal cortex and temporo-parietal junction during REM sleep, 3NREM sleep and wakefulness [49]	**X**	
	Visual dream contents are represented by discriminative brain activity patterns similar to perception in the visual cortex [57]	**X**	
**Structural neuroimaging**	Significant morphoanatomical differences in the medial prefrontal cortex between high recallers and low recallers [15,51]	**X**	
**Neurochemical**	Dopamine dosage negatively correlates with dream bizarreness and emotional load in patients with Parkinson’s disease [15]	**X**	

EEG, Electroencephalography; PSG, Polysomnography; iEEG, Intracranial-Electroencephalography; DR, dream recall; REM, Rapid Eye Movement; NREM, Non-Rapid Eye Movement.
